# “Life is hard”: How the COVID-19 pandemic affected daily stressors of women

**DOI:** 10.1016/j.dialog.2022.100018

**Published:** 2022-06-03

**Authors:** Rachel S. Purvis, Britni L. Ayers, Brett Rowland, Ramey Moore, Emily Hallgren, Pearl A. McElfish

**Affiliations:** a College of Medicine, University of Arkansas for Medical Sciences Northwest, 1125 N. College Avenue, Fayetteville, AR 72703, USA; b Office of Community Health and Research, University of Arkansas for Medical Sciences Northwest, 1125 N. College Avenue, Fayetteville, AR 72703, USA

**Keywords:** COVID-19, Stress, Women's health, Mental health, Gender

## Abstract

The COVID-19 pandemic radically and rapidly altered Americans' daily life as they navigated quarantines, school closings, job insecurity, and disrupted social activities. The COVID-19 pandemic has disproportionately affected women who have reported higher levels of stress, anxiety, and depression related to the pandemic compared to men. The study explored how the COVID-19 pandemic affected daily stressors of women. Qualitative and quantitative data were collected simultaneously using an online questionnaire from female participants (*N* = 531) who were 18 years of age or older and residing, employed, or accessing health care in Arkansas. A qualitative descriptive approach was used to summarize and synthesize participants' experiences and perceptions. Qualitative data allowed respondents to describe their lived experiences of how the COVID-19 pandemic affected them from their perspective. Four primary themes related to participants' experiences of stress related to the COVID-19 outbreak are reported: 1) employment and expenses, 2) social distancing, 3) caregiving, and 4) emotional/mental health. Several subthemes emerged within primary themes. The study documented respondents' lived experiences and how COVID-19 stress increased anxiety, depression, fear, and frustration. These findings contribute important nuances about women's experiences of stress caused by COVID-19 and can inform future health policies to address women's health post-pandemic and in future health crises. This study makes a significant contribution to the literature as the first article that uses qualitative methods to document sources of COVID-19 pandemic stress for women in their own words.

## Highlights

• Women have been disproportionately affected by the COVID-19 pandemic.
• Pandemic-related stress leads to greater anxiety, depression, fear, and frustration.
• Documenting women's experiences serves to inform health policy and practice.

## 1 Introduction

The COVID-19 pandemic radically and rapidly altered Americans' daily life as individuals navigated quarantines, school closings, job insecurity, and disrupted social activities beginning in March 2020. The disruption of daily routines has had a significant impact on the physical, mental, social, and financial well-being of the United States (US) [[1], [2], [3]] and the global population [[4], [5], [6], [7], [8]]. As the number of COVID-19 infections and deaths significantly increased in the US, acute stress and depressive symptoms related to the COVID-19 pandemic increased over time [9]. Research demonstrates that the COVID-19 pandemic has disproportionately affected women who reported higher levels of stress, anxiety, and depression related to the pandemic compared to men [1,6,8,10,11]. Specifically, younger age, female gender, and caregiver status increased the risk for COVID-19 stressor exposure and a greater degree of stressfulness [1,8].

Women who work, especially those with children, reported significantly increased COVID-19-related stress as the pandemic disrupted childcare arrangements [12]. Women have left the workforce at an alarming rate during the COVID-19 pandemic [13]. One in five (19.6%) working-age US adults report not working because the COVID-19 pandemic had disrupted childcare arrangements [14]. Of those not working, women ages 25–44 were almost three times as likely as men to not be working because of childcare demands due to the COVID-19 pandemic [14]. Studies of parental stress in the US during the COVID-19 pandemic document increased stress related to changes to children's routines, health concerns, and virtual school demands [2,12]. Working mothers with children of elementary school age or younger reported higher levels of psychological distress reflecting the gendered differences in childcare arrangements of school-age children [12,15]. The COVID-19 pandemic may have accentuated gender disparities for women since women are disproportionately taking on demands for domestic labor like childcare, virtual schooling, and eldercare [12,[15], [16], [17], [18]].

Current literature has demonstrated US women are bearing a higher burden of COVID-19-related stress [12,[14], [15], [16], [17], [18], [19], [20]]. However, no prior studies have included qualitative methods to examine COVID-19-related stress on women. Therefore, the literature lacks documentation of women's lived experiences of stress caused by COVID-19 in their own words. It is important to understand the nuanced effect of the pandemic on the daily stress of women in order to tailor interventions to effectively address gender disparities and mitigate the impact of the COVID-19 pandemic on women's mental health.

## 2 Methods

### 2.1 Study aims and design

The aim of the study presented was to explore how the COVID-19 pandemic affected the daily stressors of women. All study materials and procedures were approved by the University of Arkansas for Medical Sciences Institutional Review Board (IRB #261226). Quantitative and qualitative data were collected simultaneously with an online questionnaire [[21], [22], [23], [24], [25], [26], [27]]. Quantitative and qualitative results were integrated to capture a more complete and nuanced picture of the effects of COVID-19 on women.

### 2.2 Instruments

Survey items were selected from validated sources, including the PhenX Toolkit [28]. This toolkit is a web-based catalog of recommended measurement protocols selected by experts to include in studies with human participants [28]. The survey collected sociodemographic information, sources of pandemic-related stress, and information related to the effects of COVID-19, specifically on participants' daily lives and mental health. Sociodemographic measures included age, sex, race and ethnicity, income, and education. To capture employment status, participants were asked to identify the primary job sector for which they were employed. To examine the greatest sources of COVID-19-related stress, participants were asked, “What have been your greatest sources of stress from the COVID-19 outbreak? (Select ALL that apply.)” Participants could answer by selecting from the following response options: health concerns, financial concerns, impact on work, impact on your child, impact on your community, impact on family members, access to food, access to baby supplies (e.g., formula, diapers, wipes), access to personal care products or household supplies, access to medical care (including mental health care), access to medical supplies (e.g., prescriptions, testing supplies, etc.), and social distancing or being quarantined. Two open-ended items assessing daily stressors were used to explore participants' experiences of COVID-19-related stress and how the pandemic affected participants' daily lives. Participant experiences of COVID-19-related stress were collected by asking respondents to describe in their own words, “What other sources of stress have you experienced during the COVID-19 outbreak?” To explore the pandemic's impact on daily life, participants were asked to describe in their own words, “How has COVID-19 affected your daily life?”

### 2.3 Participant recruitment, consent, and remuneration

Potential participants were recruited from six clinical sites providing primary care throughout Arkansas between October 30, 2020 and January 16, 2021. A recruitment email was sent to potential participants inviting them to participate in a study about their perceptions and experiences related to the COVID-19 pandemic. To be included in the study, participants verified they were 18 years of age or older and working, living, and/or receiving health care in Arkansas before documenting their consent. Participants who were under 18 years of age and/or those who did not live, work, or receive health care in Arkansas were ineligible. Screening questions documenting first and last name, date of birth, street address, and email address were used to eliminate duplicate participants. Participant consent and survey responses were captured using Research Electronic Data Capture (REDCap). REDCap is a widely used web-based software created for research data capture and management [29,30]. A $20 gift card was provided as remuneration to all participants who completed the online survey.

### 2.4 Study population

A total of 876 responses to the online questionnaire were collected. Among these survey responses, a total of 809 respondents met the inclusion criteria for the study; 67 respondents were excluded because they did not meet the inclusion criteria. Among those 809 respondents who were eligible, 21 provided no information past the eligibility screener, and 34 were determined to be duplicate records and were removed. The final analytic sample included 754 respondents, of which 531 were female. These female respondents (*N* = 531) comprise the study sample.

### 2.5 Analytic strategy

Descriptive statistics were calculated for participant characteristics and perceived effects of the COVID-19 pandemic on their daily stressors. Means and standard deviations or frequencies and proportions are provided for continuous or categorical variables, respectively. A qualitative descriptive approach was utilized for data analysis of open-ended questions. A descriptive design synthesizes a summary of participants' experiences and perceptions as well as the meanings that participants attribute to them [31]. A descriptive qualitative design allows participants to express themselves with their own words. All responses were reviewed by two qualitative researchers who developed a codebook with emergent primary codes and sub codes. Data segments were coded, and confirmation-coding analysis was performed by the two primary qualitative researchers and two additional qualitative researchers. The codebook was revised three times with refined codes. Codes were categorized and organized into themes. The most illustrative quotes from the open-ended survey responses were presented under each thematic domain. Each analysis summary was critically reviewed by the research team to ensure that the data and illustrative excerpts were extracted to the appropriate thematic domain and to ensure analytic rigor and reliability. Any divergences in data interpretation were discussed by the research team and resolved via consensus. Participants provided over 500 responses to the open-ended items, which required that only the most representative quotes be presented. While quotes are presented within the themes they represent best, respondents provided descriptions of experiences and perceptions, which were often interrelated and expressed within the same sentence. Participates typed open-ended responses, and the qualitative results are presented in the grammar and punctuation participants typed to ensure their responses are reflected in their own words.

## 3 Results

### 3.1 Quantitative findings

Table 1 presents descriptive statistics of the 531 female respondents. The mean age of female respondents was 46.2 years old. More than half of respondents self-identified their race and ethnicity as non-Hispanic White (67.9%), and almost a quarter of respondents self-identified as Black or African American (20.8%). A third of respondents (32.8%) were college graduates. Almost half of respondents (45.0%) were married. While over-representative of African Americans and college graduates in Arkansas [32], the sample is diverse.

**Table 1:** Demographic characteristics of female respondents.

	Female %/mean/n
Age (years)	
Mean (s)	46.2 (15.6)
Race %	
American Indian or Alaska Native	1.7
Asian	0.9
Black or African American	20.8
Hispanic/Latino(a)	4.2
Native Hawaiian or Pacific Islander	1.7
White, non-Hispanic	67.9
More than one race	2.8
Education %	
Grades 1 through 8 (Elementary)	0.9
Grades 9 through 11 (Some high school)	4.5
Grade 12 or GED (High school graduate)	24.2
College 1 year to 3 years (Some college or technical school)	37.5
College 4 years or more (College graduate)	32.8
Marital Status %	
Single	28.2
Married	45.0
A member of an unmarried couple	5.5
Divorced/Separated	15.6
Widowed	5.6
N	531

Table 2 presents female respondents' self-reported sources of stress due to the COVID-19 outbreak. Over half of female respondents (61.0%) reported health concerns as a source of COVID-19-related stress; 62.3% reported the impact of COVID-19 on their family members, and 33.0% reported the impact of the pandemic on their child as sources of stress. A third (34.8%) of female respondents reported the impact on your community as a source of COVID-19-related stress. Approximately half of respondents (54.2%) reported stress related to social distancing or being quarantined. Almost half of female respondents (47.3%) reported financial concerns as a source of pandemic-related stress, and 41.8% of female respondents reported the impact on work as a source of stress due to the COVID-19 outbreak.

**Table 2:** Sources of stress due to COVID-19.

	%
Impact on your family members	62.3%
Health concerns	61.0%
Social distancing or being quarantined	54.2%
Financial concerns	47.3%
Impact on work	41.8%
Impact on your community	34.8%
Impact on your child	33.0%
Access to personal care products or household supplies	28.1%
Access to medical care, including mental health care	25.8%
Access to food	23.4%
Access to medical supplies (e.g., prescriptions, testing supplies)	16.8%
Access to baby supplies (e.g., formula, diapers, wipes)	4.1%
Other	3.4%
I am not stressed about the COVID-19 outbreak	3.0%
Don't know/Not sure	0.2%
N	531

*Note:* Percentages do not total 100 as respondents were asked to select all that apply.

Among the 531 female respondents, 438 (82.5%) reported being employed prior to the COVID-19 pandemic. Table 3 presents the primary job sector female respondents reported working in prior to the pandemic. The most commonly reported job sectors were health care (16.9%) and school/education system (14.2%). A small portion of respondents (20.8%) selected “other” as their job sector and were asked to specify. Job sectors specified in the “other” category included child care/daycare worker, airline employee, and food delivery driver.

**Table 3:** Primary job sector of female respondents.

	%
Other	20.8%
Health care worker (e.g., doctor, nurse, nursing aide) or first responder	16.9%
School/education system (as employee not student)	14.2%
Don't know/Not sure	7.3%
Clerical support worker (office clerk, secretary, customer service clerk)	6.6%
Service and sales worker (travel agent, cook, hair dresser/barber, retail sales, cashier)	5.9%
Work in a clinic or hospital but not as a health care worker	5.0%
Student	4.3%
Prefer not to answer	4.1%
Professional (physical/earth science/engineering professional, business/sales and marketing professional, software developer, legal, author, journalist, performing artist)	4.1%
Cleaning or maintenance worker occupations (cleaner, helper, agricultural laborer, transport laborer, street vendor, refuse worker)	2.5%
Manager (chief executive, administrative manager, production and sales, hospitality and retail manager)	2.3%
Plant and machine operator and assembler (includes truck drivers)	1.8%
Faith	1.6%
Technician or associate professional (in field of engineering, business, legal, social, or information/communications)	1.1%
Work in a food processing plant	0.9%
Craft and related trades worker (builder, machinist, electrician, printing)	0.5%
N	438

Table 4 presents the self-reported effect of the COVID-19 pandemic on participants' work. Approximately one-quarter of all female respondents (23.4%) reported their job put them at increased risk for getting COVID-19. Thirty female respondents (5.6%) reported they lost their job permanently, 26 (4.9%) reported they lost their job temporarily, and 62 (11.7%) reported their work hours were reduced.

**Table 4:** Effects of COVID-19 pandemic on work.

	%
None of these apply	24.5%
My job put me at increased risk of getting COVID-19	23.4%
I did not have a paying job before the COVID-19 outbreak	14.5%
I reduced my work hours	11.7%
I increased my work hours	7.5%
I got a new job	6.6%
I lost my job permanently	5.6%
I lost my job temporarily	4.9%
Prefer not to answer	1.7%
Don't know/Not sure	1.1%
I laid off employees	0.8%
N	531

*Note:* Percentages do not total 100 as respondents were asked to select all that apply.

### 3.2 Qualitative findings

Four primary themes related to sources of stress that participants experienced during the COVID-19 outbreak emerged. Respondents reported 1) employment and expenses, 2) social distancing, 3) caregiving, and 4) emotional/mental health as sources of COVID-19-related stress. Subthemes emerged within each primary theme. Within respondent descriptions of employment and expenses as sources of stress, three subthemes were identified; these subthemes included essential/frontline workers, employment disruptions, and behind on rent or bills. For respondent descriptions of social distancing as a COVID-19 source of stress, three subthemes emerged. These included limited to no social engagements outside of immediate family members, canceled travel, and canceled social activities. Within respondent descriptions of caregiving stress, three subthemes related to work-life balance, childcare/virtual learning, and elder care emerged. Four subthemes were identified within respondents' descriptions of their emotional/mental health that included increased anxiety, depressed, fear, and frustration. Table 5 presents emergent themes and subthemes.

**Table 5:** Emergent themes and subthemes related to sources of stress for female respondents during the COVID-19 pandemic.

Theme	*Subthemes*
Employment and expenses	*Essential/frontline workers* *Employment disruptions* *Behind on rent and bills*
Social distancing	*Limited to no social engagements outside of immediate family members* *Canceled travel* *Canceled social activities*
Caregiving	*Work-life balance* *Childcare/virtual learning* *Elder care*
Emotional/mental health	*Increased Anxiety* *Depressed* *Fear* *Frustration*

### 3.3 Employment and expenses

Respondents reported the effect on their employment and expenses as primary sources of stress related to the COVID-19 pandemic. Within the quantitative data, almost half of respondents (41.8%) reported the pandemic's “impact on work” as a source of stress, and almost half (47.3%) reported “financial concerns” as a source of stress due to the COVID-19 outbreak. In response to open-ended survey items, respondents identified being an *essential/frontline worker*, *employment disruptions*, and being *behind on rent and bills* as the main sources of stress they had experienced related to the COVID-19 pandemic.

#### 3.3.1 Essential/frontline workers

About a third of female respondents indicated their primary job sector as either health care (16.9%) or education (14.2%), which were essential/frontline worker sectors during the COVID-19 pandemic. In open-ended responses, respondents identified also being an essential/frontline worker as the primary source of COVID-19-related daily stress. One respondent explained, “I am considered an essential worker; therefore, I have had to work constantly throughout the past few months. Which is very tiresome.” Frontline workers reported the emotional toll of their job during the pandemic. A respondent who is a nurse said, “Work is stressful and disheartening. I have lost coworkers, seen young people die.” Another reported, “I work in a nursing home. It's just sad and heartbreaking to watch our residents cry and become more withdrawn because they miss their families so much. It's hard.” Other respondents explained how adapting their job function was a source of stress. A public high school teacher said, “COVID-19 has impacted my daily routine, professional procedures, and greatly increased my workload.” Another respondent said, “While our school has maintained the same number of daily and weekly hours for onsite instruction, my workload and time spent on professional tasks have increased significantly.”

#### 3.3.2 Employment disruptions

Quantitative responses showed that approximately a quarter of female respondents (22.2%) had permanently lost their job, temporarily lost their job, or had their work hours reduced because of the COVID-19 pandemic. Respondents also described a reduction in hours, losing employment, or retiring earlier than planned resulting in lost income. Respondents said, “I am currently working full time but for a month, my place of employment reduced hours for 30 days,” and “Working under these standards have affected my tremendously. With cut hours and everything is stressful.” Other respondents were laid off or decided to retire early, which affected their income and was a source of stress related to the pandemic. One respondent shared, “I lost my second full time job due to covid. I've lost out on $12,000 take home.” Another respondent explained, “My son who has, type one diabetes, and I have been under an isolation order since March. I took FFCRA leave and all of my sick leave to protect my son. Eventually, I have been forced to retire a year and a half ahead of schedule. My income will be third of what it was.” Other respondents describe temporary disruptions based on the need to quarantine. One respondent explained, “I actually had COVID-19. I didn't have the worst of it but it put me out of work for a month.” COVID-19 mitigation protocols, like quarantining after exposure or infection, affected respondents' work hours. A respondent described, “It caused hardship on my family. I am single mom and when my kids or I get quarantined, I lose money.”

#### 3.3.3 Behind on rent and bills

Almost half of female respondents (47.3%) reported “financial concerns” as a source of stress due to the COVID-19 pandemic. Interrelated to stress about financial concerns like losing their job or having a reduction in employable hours, respondents described being behind on paying their living expenses like rent and utilities as a great source of COVID-19-related stress. One respondent explained, “I am out of work, all my bills are way behind, I'm about months behind in rent and I'm terrified for my family and self.” Respondents said they were “stressed about bills,” and “life is hard and my family struggles with bills and stresses over these things.” Other respondents were stressed about being behind on their bills because they had lost second jobs or supplemental income because of the COVID-19 pandemic. A respondent explained, “I lost all of my part-time/supplemental income sources with the onset of the pandemic. My financial situation has yet to recover and therefore the strain on my budget impacts my day to day life in countless ways.” Another respondent said, “I was denied all unemployment and pandemic assistance for losing my second full time income, which has not allowed me to pay water or energy bills since late March and I'm still past due on rent.”

### 3.4 Social distancing

Respondents reported social distancing as a main source of stress related to the COVID-19 pandemic. Over half of respondents (54.2%) reported “social distancing or being quarantined” as a source of stress due to the COVID-19 pandemic. A third of respondents (34.8%) reported “impact on your community” as a source of stress due to the COVID-19 pandemic. When describing the stress of social distancing, respondents described having *limited to no social engagement outside of immediate family members, canceled travel,* and *canceled social activities*.

#### 3.4.1 Limited to no social engagements outside of immediate family members

Respondents reported having limited to no social interaction or engagement outside of their immediate family members as a source of COVID-19-related stress. A respondent explained, “My relationship are worse, my screen time is through the roof, and I mostly only see or talk to the people I live with.” Another respondent said, “I do not socialize with friends in person anymore and the only people I see outside of work are close family members.” A respondent reported, “I miss seeing my extended family for game night.” One respondent described, “It's been hard to be home alone and not have family or friends visit. I feel extremely isolated. I haven't been able to even hug my mom in more than six months.” Another respondent said:

We do not eat out anymore, sometimes we pick up to go, but even that seems dangerous unless you know the restaurant is enforcing the proper COVID procedures. I no longer go to the nail salon, no movies, no social gatherings, etc. Pretty much my life is at home with my immediate family. I could really use a vacation, but do not want to risk it.

#### 3.4.2 Canceled travel

Respondents explained that social distancing was a source of stress because they were unable to travel to visit family, friends, and work colleagues, resulting in loneliness and isolation. Respondents said, “We have not been able to travel to see relatives abroad or go out to town often to see extended family in another state due to safety concerns,” and “no travel or vacations [and] very limited gatherings with friends and family.” Another respondent reported they are “unable to travel to see family or work team as much, unable to hug or touch my elderly parents, and unable to social in person, [and have a] depressed mood.” Other respondents echoed the stress of canceled travel, stating, “It just makes me a little sad that we don't get to travel for important events,” “cannot enjoy family vacations,” and “did not go anywhere on vacation this summer.”

#### 3.4.3 Canceled social activities

Respondents also reported that social distancing was a source of stress because it meant many social activities that relieved stress were canceled to try to stop COVID-19 from spreading. A respondent explained, “I miss seeing my monthly Bunko group. I miss getting my nails done. Things that would normally relieve stress and help me relax I no longer do.” Respondents reported canceled worship services saying, “We do not attend church. We watch our church services live online,” and “it has stopped my small group activity with my church and stopped in person worship.” Another respondent said, “COVID-19 caused rugby to be canceled. Rugby is a major part of my life. It is a stress reliever, it is how I see my friends, and it is a key to my weekly routine. With rugby being canceled I have had to find new ways to cope with stress and find creative ways to see my friends.”

### 3.5 Caregiving

Respondents described caregiving responsibilities as a major source of stress related to the COVID-19 pandemic. Three subthemes emerged related to caregiving stress (see Table 5). A third of respondents (33.0%) identified “impact on your child” as a source of stress due to the COVID-19 outbreak, and almost two-thirds of respondents (62.3%) reported “impact on your family members” as a source of pandemic-related stress. In response to open ended items, respondents described *work-life balance, childcare/virtual learning,* and *elder care* as primary sources of stress related to the COVID-19 pandemic.

#### 3.5.1 Work-life balance

Respondents explained that maintaining a work-life balance was a major source of COVID-19-related stress. One woman succinctly explained, “At the beginning it felt nice to be with immediate family but then that became stressful when work from home/family balance got difficult to maintain.” Others said, “I feel like I need to work and take care of household responsibilities during the day which overloads me,” and “It has made it more difficult to have a job outside of the home and to get my kids into daycare and school.” Another respondent stated, “My husband lost his great paying job due to COVID and that has forced me to go back to work and attend school at the same time along with taking care of my family, which has put alot of stress on me.” Respondents explained caregiving included magining the stress and depresson of their families:

My son's grades have lowered due to him not being in class and having everyday human interaction. Days that just feel lazy and no energy. My mother's depression has increased because she can't do activities outside the home. As a caregiver this makes it very hard to manage everyone's feeling and on top of all that work from home.

#### 3.5.2 Childcare/virtual learning

Respondents identified new childcare and virtual learning demands as causes of COVID-19-related daily stress. Respondents said, “My kids [school] are all virtual so it's stressful,” and “almost our whole day is trying to get through mountains of tortuous online homework.” Balancing childcare responsibilities with virtual learning was also stressful as one respondent explained: “I have had to become a homeschool teacher for my oldest daughter, which is extremely hard with two younger children not yet in school [so] it's quite stressful.” Another respondent said, “Any energy I have that would normally go to getting to work part time is consumed by having my kids at home full time and helping them with their online school full time.” A respondent who was unemployed and unable to balance virtual learning and job hunting explained, “Because my daughter is home for virtual school I am not able to look for work at this time.”

#### 3.5.3 Elder care

Another source of stress that respondents discussed was caring for elderly parents and the fear that elderly family members in their care may get COVID-19. One respondent stated, “My elderly father lives with me and he has COPD and it has been very stressful worrying about him catching it [COVID-19] from me.” An essential worker said, “I work in health care and also take care of my elderly and ill mother [and] working during a pandemic adds anxiety and stress to my daily life as I am hyperaware of the effects the virus could have on my family if I were to get sick.” Others described the precautions they had undertaken to limit exposing elders in their care. One respondent said, “My elderly parents live with us so we have to take extra precautions and limit our activities and exposure during this time.” Another respondent explained, “I have lost one job and had to quit another due to COVID-19 [because] I live near and have lots of contact with my elderly parents and can't take the risk of getting them sick.”

### 3.6 Emotional/mental health

Interrelated to the sources of COVID-19-related stress, respondents explained how these stressors affected their emotional and mental health. Four subthemes were identified (see Table 5). In their open-ended responses, respondents described having *increased anxiety,* feeling *depressed, fear,* and *frustration* when discussing the impact of COVID-19-related stress on their emotional and mental health.

#### 3.6.1 Increased anxiety

Respondents stated they experienced increased anxiety due to the pandemic and its impact on their family, community, and society and explained they were worried, stressed, and concerned. One respondent explained, “Don't know how to express it but . . . I feel an overall anxiety and sense of void in not being able to touch my family and other human beings. It's unnatural and abnormal as we all know.” A respondent reported, “My mental health has been somewhat affected by the general increase in concerns for my friends, family, community, nation, and myself.” Another respondent said, “I do feel a general anxiety about COVID-19 and the impact it will have on my family and others around the world.” Other respondents said, “Very stressed out concerned about family and concerned for my community,” and “worry about friends and family members who are subject to exposure at their work places or other community venues (grocery stores, church, pharmacies, etc.) even if those places comply with CDC guidelines.”

#### 3.6.2 Depressed

Respondents reported feeling sad, isolated, and lonely due to social distancing and quarantine stress. A respondent explained the “added stress [being] stuck at home, feelings of sadness and uselessness on some days.” Another respondent described “feelings of being stuck, sadness, occasional feelings of needing to do things but no energy or motivation to do them.” Other respondents said, “I live alone so this is very lonely at times,” and “sometimes I feel depressed and down in the dumps [and] I have to take breaks from the news and Instagram because it can be overwhelming.” Feelings of depression and anxiety were interrelated for most respondents. For example, one respondent said, “I am sad and depressed, I feel hopeless and anxious about my personal situation and the state of the country.”

#### 3.6.3 Fear

Respondents reported being afraid of contracting COVID-19 or exposing loved ones to COVID-19. A respondent stated, “I live with a lot of fear of getting sick and dying.” Another respondent explained that their “fear was so strong that I left my job as a clinic nurse and found a job that gave me very limited exposure to others.” Other respondents said, “Some places I just don't go to because of fear,” and “due to fear of COVID I have canceled doctor's appointments after getting to the office and it was too crowded for me.” Another respondent explained, “I'm afraid to leave my house for fear of getting it.” A respondent described a recent habit of daily outdoor walks meant she had to overcome her fear: “I just started doing this as I was even afraid to go out at all for the longest time.”

#### 3.6.4 Frustration

Respondents described frustration with both how individuals were adhering to COVID-19 mitigation efforts (i.e., wearing masks, social distancing, etc.) and government response at the local, state, and national levels. As one respondent explained, “how the federal government has dealt with this deadly disease highly distresses me.” Another respondent said, “I feel relatively unseen and unprotected by the policies being put in place by the state; as a childcare provider I have received very little in the way of education, guidance, or other support. I am constantly afraid that my school with have to close down and I will lose income.” Other respondents reported frustration with individuals. One respondent said, “It has made me more frustrated with people I don't even know, I feel upset when I see people not wearing a mask, not social distancing, spreading misinformation, etc.” Another respondent explained they had “frustration that other people's irresponsible decisions are negatively effecting us all (including killing people) and frustration with poor government leadership in this crisis, which I feel is prolonging the effects and length of this pandemic nationwide.”

## 4 Discussion

This study documents respondents' lived experiences with stressors due to the COVID-19 pandemic using quantitative and qualitative methodology. Respondents reported *employment and expenses, social distancing,* and *caregiving* as primary sources of COVID-19-related stress. These findings are consistent with studies that have documented the COVID-19-related stressors most commonly reported among the US population include stress over the spread of COVID-19 infection, uncertainty and disruption of normal daily routines, and financial concerns [1,2,12]. Respondents in this study described the effect of these COVID-19 stressors on their *emotional and mental health*, reporting increased anxiety, depression, fear, and frustration. These findings support previous research that shows women, especially those with caregiving responsibilities, are more likely to experience greater stress due to the COVID-19 pandemic [1,12,15]. This article makes a significant contribution to the literature as the first study to document how COVID-19 has increased the stress of US women in their own words.

Some respondents explained they experienced increased stress due to the COVID-19 pandemic because their profession was classified as essential/frontlines. Respondents described the emotional and physical stress of their job during the COVID-19 pandemic including sadness and exhaustion. These findings are consistent with prior research that found essential workers such as those in health care were more likely to experience anxiety, depression, post-traumatic stress disorder, and sleep disorders [33]. Employment-related stress was interrelated to respondents' concerns about job security and being able to pay their living expenses, which is consistent with the literature that has documented financial concerns as a primary stressor related to COVID-19 [1]. Respondents' descriptions of stress related to social distancing, quarantine, and other mitigation measures supports the literature documenting the stress related to the pandemic's disruption to daily routines such as working from home, virtual learning, and childcare centers closing [1,2,12].

In their quantitative responses, almost two-thirds of female respondents identified the impact on their family members as a source of COVID-19-related stress. Respondents' qualitative responses provided further details about how respondents characterized this impact with their descriptions of the stress of maintaining a work-life balance while providing childcare, especially for school-aged children who were attending school virtually. These findings support the previous literature, which has documented gendered differences in household responsibilities like childcare during COVID-19 [2,12]. Respondents described how COVID-19-related stress increased their anxiety and depression, which is consistent with research documenting the effect of the pandemic on women's mental health [9,18]. Respondents reported the fear they experienced related to the COVID-19 pandemic and how it was connected to an increase in their stress. Respondents identified being frustrated with how individuals were not adhering to mitigation requirements like mask wearing and the governmental response to the pandemic; respondents directly connected this frustration with an increase in their stress due to COVID-19. This is the first literature to document these findings in participants' own words. These findings provide important nuanced insight on participant experiences that support the national trends related to COVID-19 stressors documented in the US population [1,2,9,12]. Importantly, this study demonstrates how interrelated these stressors are. As women documented their stressors in their own words, many of the themes and sub-themes overlapped.

## 5 Limitations

This study is not without limitations. Participants were recruited from six clinics across Arkansas and may not be representative of the general Arkansas or US adult populations. While participants reported a higher level of educational attainment than the state average, the sample is racially and ethnically diverse with a slight overrepresentation of Black participants. The open-ended survey items provided participants with the opportunity to report anonymously their stressors in their own words; however, researchers did not have the opportunity to probe participants for clarification of their written responses. Finally, the study presents cross-sectional findings of COVID-19-related stressors that may not capture the variation in stress as the COVID-19 pandemic progressed.

## 6 Conclusions

This study makes a significant contribution to the literature by documenting sources of stress for women caused by the COVID-19 pandemic using both quantitative and qualitative methods. This study documents how the COVID-19 pandemic affected the daily stress of women in their own words. These findings contribute important nuances about women's experiences and perceptions of stress caused by COVID-19, which can better inform future health policies to address women's health such as greater access to mental health services, childcare and parental support services, and greater flexibility to work from home. Further research is needed to determine the long-term mental health and employment effects for women in the US post-COVID-19. It is important to understand the effect of the pandemic on the daily stress of women in order to tailor interventions to address gender disparities and mitigate the impact of the COVID-19 pandemic and future pandemics on women's mental health. This is particularly important as the pandemic shut downs continue to impact women's employment [34].

## Declaration of Competing Interest

The authors declare no conflict of interest.
